# Evaluating the performance of a video expert panel in assessing respiratory rate from recorded videos for the diagnosis of non-severe paediatric pneumonia

**DOI:** 10.1136/bmjresp-2025-003591

**Published:** 2025-12-17

**Authors:** Ahad Mahmud Khan, Md Shafiqul Islam, Nabidul H Chowdhury, Salahuddin Ahmed, Ting Shi, Abdullah H Baqui, Steve Cunningham, Eric D McCollum, Harry Campbell

**Affiliations:** 1 Projahnmo Research Foundation, Dhaka, Bangladesh; 2 Usher Institute, The University of Edinburgh, Edinburgh, Scotland, UK; 3 Department of International Health, Johns Hopkins University Bloomberg School of Public Health, Baltimore, Maryland, USA; 4 Centre for Inflammation Research, The University of Edinburgh, Edinburgh, Scotland, UK; 5 Department of Paediatrics, Johns Hopkins School of Medicine, Baltimore, Maryland, USA

**Keywords:** Pneumonia

## Abstract

**Objective:**

To describe the process of interpreting chest movement videos for respiratory rate (RR) assessment in children aged 0–59 months with suspected non-severe pneumonia by a video expert panel (VEP) and to evaluate the panel’s performance.

**Methods:**

Six physicians trained and standardised to RR assessment from recorded videos formed a VEP. The panel interpreted RR from recorded videos collected from health facilities in Bangladesh. The videos were distributed using a web-based system among the panel members. Each member was evaluated by assessing inter-reader agreement among the primary readers, each reader’s agreement with the final panel reading and intrareader agreement using per cent agreement. Agreement was defined as the video being interpretable and the difference in RR count between two readers being ≤2 breaths per minute. The panel’s performance was evaluated using Bland-Altman analysis against a panel of paediatricians who reviewed a randomly selected 10% subset of videos.

**Results:**

Among the 605 videos, the VEP classified 89.9% (n=544) of them as interpretable with RR count difference ≤2 breaths per minute, 2.0% (n=12) as interpretable with RR count difference >2 breaths per minute and 8.1% (n=49) as uninterpretable. For the video interpretability, inter-reader agreement among primary readers ranged from 88% to 95%, each reader’s agreement with the final panel was 90%–95%, and intrareader agreement was 98%–100%. For the RR measurement, five out of six VEP members demonstrated satisfactory inter-reader agreement (80%–83%), agreement with final panel reading (92%–97%) and intrareader agreement (92%–100%). One outlier reader showed lower agreement levels of 55%, 61% and 75%, respectively. The mean difference in RR counts between the VEP and paediatrician panel was −0.3 breaths per minute, and limits of agreement were −3.0–2.3 breaths per minute.

**Conclusion:**

The performance of VEP was satisfactory for evaluating RR counting. The study provided valuable insights into the development and evaluation of the VEP as a potential reference standard.

**WHAT IS ALREADY KNOWN ON THIS TOPIC**

- There is no established ‘gold reference standard’ for evaluating respiratory rate (RR) counting in the diagnosis of paediatric pneumonia. A video expert panel (VEP) could be an improved reference standard for use in research studies.

**WHAT THIS STUDY ADDS**

- The study described a process of developing the VEP and evaluated the performance of individual panel members and overall panel.

**HOW THIS STUDY MIGHT AFFECT RESEARCH, PRACTICE OR POLICY**

- This study has established a foundation for future research to refine and validate the VEP approach, contributing to the development of an ideal reference standard for RR counting.

## Introduction

 Pneumonia is a leading cause of mortality in children under 5 years old worldwide.^1^ The global incidence rate of pneumonia is 0.11 (0.09–0.13) episodes per child-year.^2^ In 2019, there were 45 million cases of pneumonia and 0.7 million deaths attributed to it.^3^ In low- and middle-income countries (LMICs), millions of pneumonia cases occur annually, with the majority of deaths also concentrated in these regions. The highest burden is observed in sub-Saharan Africa, South Asia and Southeast Asia.^4^

The WHO Integrated Management of Childhood Illness (IMCI) guidelines diagnose pneumonia primarily by counting respiratory rate (RR).^5 6^ Frontline health workers in LMICs observe the child’s chest, manually count RR and identify age-specific fast breathing.^7^ This method relies on the observer’s capability to count RR accurately, making it susceptible to errors.^8 9^ Observing chest movements while simultaneously counting RR using a timer or watch is challenging. Some may also face difficulties remembering the count.^10^ Critically, studies have shown that many health workers do not count RR at all, instead relying on subjective judgement or clinical impression.^11^ This often leads to the misclassification of fast breathing, resulting in incorrect pneumonia diagnosis and inappropriate treatment.^12^

Automated RR counters are primarily used for continuous monitoring of RR in high-resource settings, and these tools are highly technological.^10 13^ This improved technology for counting RR has not yet been adopted in low-resource settings. The availability of improved diagnostics to support frontline health workers might improve the diagnosis of pneumonia. The UNICEF introduced the acute respiratory infection diagnostic aid project and shared a target product profile (TPP) for guiding the manufacturing of new automated RR counting devices.^14^ Based on this, some automated RR counting devices have been developed suitable for use by frontline health workers, for example, Children’s Automated Respiration Monitor (ChARM),^15 16^ Rad-G,^17 18^ uPM60,^19^ Respimometer.^20^

From the methodological point of view, when potential new technologies are introduced, their performance should be evaluated using a robust and established method in a consistent and generalisable way. The absence of an appropriate reference standard to evaluate RR counting is challenging.^21^ Most have relied on trained medical professionals (eg, physicians, nurses) to measure the RR manually as the reference standard. However, manual RR counting is often considered unreliable.^10^ Potential biases with human counts include difficulties in measuring RR over the same period and inconsistencies in counting.^21 22^ Some studies have used automated monitors as the reference standard for RR.^23 24^ These methods also have biases since the automated monitors measure variables, such as carbon dioxide, pulse oximeter signal or sound to extract RR, rather than directly observing chest movements.^1025^,^27^ The concern regarding reference standards has recently become a topic of discussion in the literature.^21^

The use of video recording can be helpful in respiratory research. Video recording of child chest movements and subsequent interpretation of RR by two paediatricians was done in a study in Tanzania by Muro *et al.*^28^ Sinyangwe *et al* also performed videography of child chest movements in a study conducted in Zambia for validating RR count by community health workers.^29^ Videography of child chest movements and using experts to assess RR from the recorded videos could be a potential solution to reference standard. However, in diagnostic accuracy studies, getting an accurate final diagnosis for each subject is often challenging because of the unavailability of a single and error-free method as the reference standard.^30 31^ There might be variability in the RR counts among the experts when interpreting RR from video recordings.^28^ A video expert panel (VEP) adjudication, which could assess a patient’s RR based on video footage to generate a final reference RR, would be an ideal reference standard for evaluating RR counting in research studies. However, there is no guidance on the preferred methodology for developing an expert panel.^32^ Stratil *et al* developed a panel of reviewers and conducted an inter-rater reliability study to evaluate the reviewers in counting RR using a video-based reference tool.^33^ If quality videos could be captured and the interpretation of RR from these videos could be systematically conducted by a VEP, it could be an ideal and non-biased reference standard.

In this report, we described the systematic approach used by the VEP to evaluate chest movement videos for accurate RR measurement in children aged 0–59 months with suspected non-severe pneumonia. We outlined the methods used by the VEP to ensure consistent and reliable RR assessments across different cases, providing insight into the processes undertaken to standardise evaluations. Additionally, we analysed the performance of individual VEP members, as well as the overall performance of the VEP as a collective unit, to assess their accuracy and reliability in RR measurement. This evaluation offers a comprehensive understanding of the VEP’s ability to serve as a reference standard for RR counting in research studies.

## Methods

### Study setting

We conducted this study in 2021–2022 at different levels of health facilities in Bangladesh. These included the outpatient department of a subdistrict hospital (Zakiganj Upazila Health Complex) and three community clinics (CCs) in Sylhet as well as the inpatient department of Institute of Child and Mother Hospital (ICMH) in Dhaka. The methodology of the study is detailed in a separate publication.^34^

### Chest videography eligibility

Study physicians at hospitals and community healthcare providers (CHCPs) at CCs clinically evaluated children for chest videography eligibility according to a standardised study protocol. Children under 2 months of age with any illness and those aged 2–59 months with cough or difficulty breathing who presented to the hospital or the CCs, provided written informed consent, had their RR counted and underwent chest videography. Children presenting with any danger sign, that is, unable to drink or feed, vomiting everything, having convulsions, lethargic or unconscious, were excluded.

### Chest video image acquisition and editing procedures

A comprehensive description of the videography procedure is provided elsewhere.^35^ Chest videos were captured during RR measurement twice for each child. A research staff recorded chest videos using a Canon EOS M50 camera.^36^ If natural light was judged insufficient, that is, the child’s chest movements were not clearly visible on the camera screen, a light-emitting diode lamp was used for additional lighting. The child’s clothing was removed from the lower neck to the umbilicus to expose the chest and belly, ensuring no identifiable parts, like the face, were recorded.

Once the child was calm—defined as not crying, moving excessively or showing visible distress for at least 30 s—the research staff began video recording of chest movements and the physician or CHCPs tapped the microphone to signal the start of counting RR. The physician or CHCPs counted RR for 1 min, tapped the microphone again to indicate completion, and the recording was stopped. Subsequently, the physician or CHCPs attached the ChARM device, and chest video recording started. Once the child was calm, the physician or CHCPs switched on the ChARM device, and the video recording proceeded concurrently with the use of the ChARM, as previously conducted. The ChARM device signalled completion with either a green or red signal, and the recording continued until the ChARM finished its count.

Each video file was initially saved on the camera, then transferred to a password-protected computer. Subsequently, they were edited to remove excess footage, eliminate sound and identifiable features and reduce file size using a video editing software named Adobe Premiere Pro.

The details of the chest movement videography and editing procedure are provided in the supplementary material.

### Training and standardisation to develop VEP

We created some reference videos for training purposes. For this, we recorded videos of 50 children from ICMH. We had three paediatricians who held postgraduate degrees in paediatrics, and each video was assessed by two random paediatricians. If there was disagreement in interpretability or a difference in RR exceeding 2 breaths per minute, the video was transferred to the third paediatrician. Videos where two paediatricians agreed on interpretability and had a RR difference of ≤2 breaths per minute were designated as reference videos. The final RR count was determined as the mean of the RR counts provided by the paediatricians in agreement.

In addition, we selected six physicians with a Bachelor of Medicine and Bachelor of Surgery degree for the training. They were trained to count RR using reference videos of known RR counts. The training was conducted by the principal investigator (study PI, AMK). The WHO IMCI guidelines were followed to provide the training.^6 37^ After the training, each physician was assigned the same 20 reference videos with varying RR. The RR counted by each physician was considered correct if it fell within ±2 breaths per minute of the RR determined by the panel of paediatricians. All six physicians achieved a passing score of 80% or above in the evaluation and were qualified to serve as VEP members in the study. The details of the training and standardisation process have been described elsewhere.^38^

### Chest video interpretation procedures

**Figure 1:**
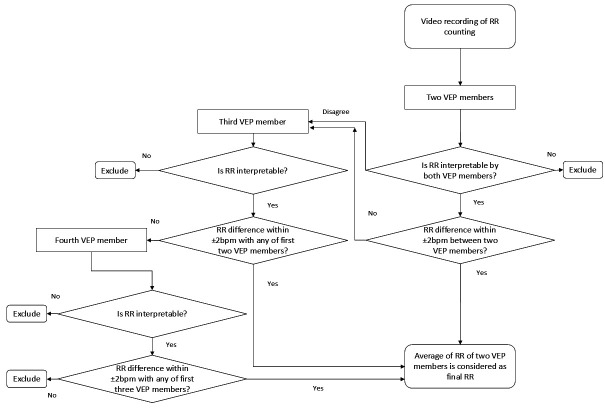
Respiratory rate interpretation process from recorded videos by video expert panel. RR, respiratory rate; VEP, video expert panel.

RR interpretation procedure from video recordings is shown in figure 1. Each video was randomly assigned to two VEP members (primary readers). Videos deemed uninterpretable by both members were excluded. If the RR difference between the two members was within 2 breaths per minute, their average RR was considered final. In cases of disagreement on interpretability or an RR difference >2 breaths per minute, the video was reviewed by a third panel member. If inconsistencies persisted (ie, no two members reported an RR difference of ≤2 breaths per minute), the video was sent to a fourth panel member. If the fourth member’s interpretation aligned with any previous member (RR difference ≤2 breaths per minute), their average RR was considered final. Videos without agreement between any two panel members were excluded from the analysis. A password-protected web-based system was used for the automated distribution of the videos among the VEP members.

### Operational definitions

*Agreement in RR counts:* defined as a difference of ≤2 breaths per minute between two readers, consistent with the most commonly used definition in the existing literature.^23 29 33 39 40^

*Concordant interpretation:* the concordant interpretation was defined when two VEP members interpreted the video as:

- uninterpretable.
- interpretable and the RR count difference was ≤2 breaths per minute.

*Discordant interpretation:* A discordant interpretation was when

- one VEP member interpreted the video as interpretable and another interpreted it as unreadable.
- the RR count difference was >2 breaths per minute.

### Chest videography and interpretation quality control measures

The study PI (AMK) periodically reviewed the videos to ensure quality and provided corrective feedback as needed. The panel of three paediatricians independently reviewed a random 10% sample of videos, blinded to the VEP’s final RR, to evaluate the VEP’s performance. The paediatricians functioned independently to validate the performance of VEP. The interpretation methodology for the paediatrician panel was the same as that of the VEP. Additionally, to assess the reproducibility of individual panel member interpretations, each member was randomly reassigned 20% of the videos they had previously interpreted.

### Measures of VEP performance

*Interobserver agreement between primary readers:* agreement between each reader’s interpretation and those of other readers for the same set of videos during the initial round of assessment.

*Agreement between individual reader and the VEP:* agreement between each reader’s interpretation and the final VEP interpretation.

*Intrareader agreement:* agreement between a reader’s interpretations when evaluating the same video on two separate occasions.

*Agreement between VEP and paediatricians:* agreement between the VEP’s final interpretation and the interpretation of the paediatrician panel.

### Statistical analysis

We assessed inter-reader agreement in interpretability and RR counts within 2 breaths per minute among primary VEP members by calculating the per cent agreement. Agreement between individual VEP members and the final panel RR count was similarly evaluated. Intrareader agreement was determined using per cent agreement for each VEP member when reassessing videos. Per cent agreements were presented with 95% CIs. The agreement between the VEP and panel of paediatricians was analysed using a Bland-Altman plot, along with calculating the per cent agreement within 2 breaths per minute between the two groups. Additionally, factors associated with the interpretability and agreement of RR counts were explored using univariable analyses (χ^2^ or Fisher’s exact tests) and multivariable logistic regression. The logistic regression model included variables if their p value was <0.2 in the bivariable analysis. P value <0.05 was considered statistically significant. All the analyses were performed using the Stata V.17.0 software.

## Results

**Figure 2:**
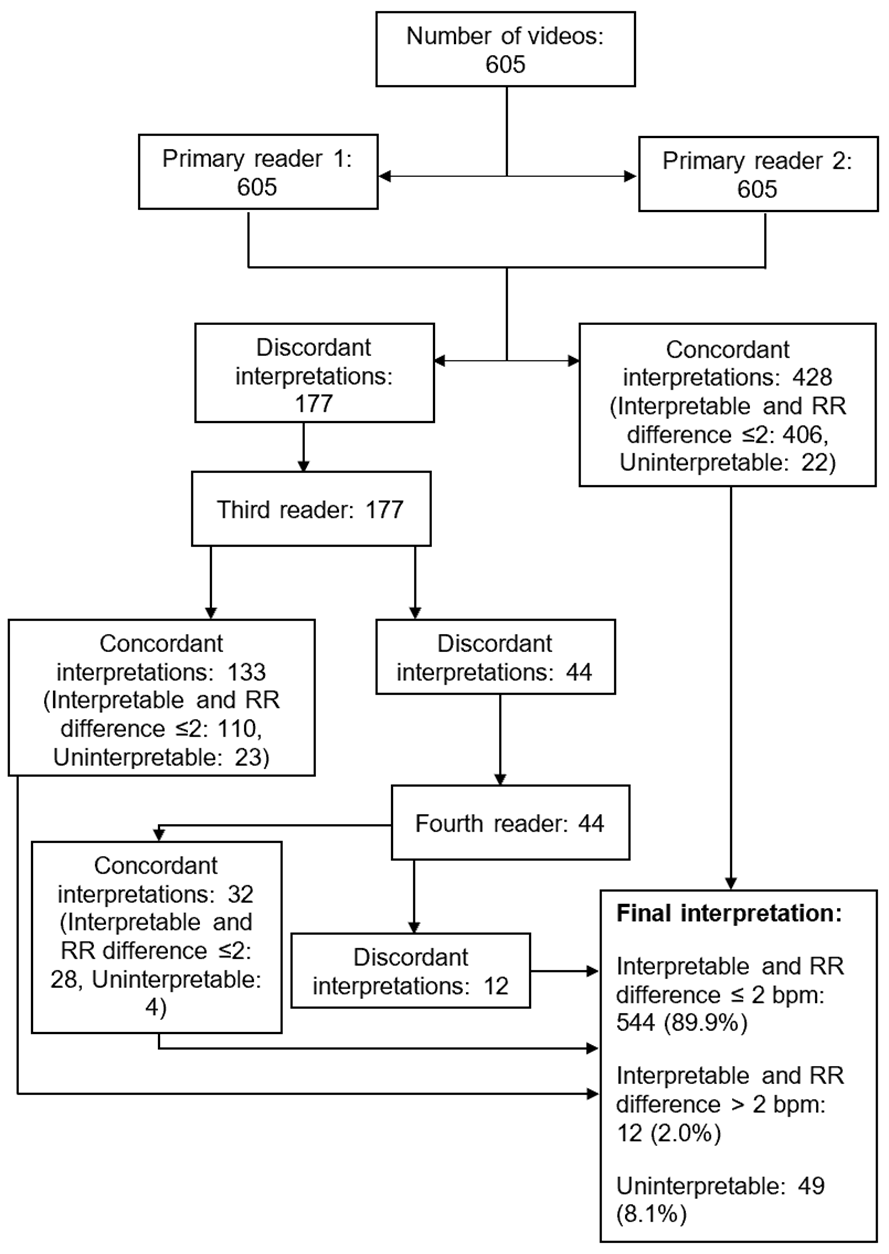
Chest video image interpretation schema. RR, respiratory rate.

A total of 612 children were screened, 340 fulfilled the eligibility criteria and 339 were enrolled. RR was manually counted from 311 children and a total of 605 videos were recorded and interpreted by the VEP. Of the 605 recorded videos, 428 (70.7%) were interpretable with an RR difference within 2 breaths per minute by the two primary readers, while 22 (3.6%) were deemed uninterpretable. Among the remaining 177 videos not agreed on by the primary readers, 110 (62.1%) were interpretable with an RR difference within 2 breaths per minute between a primary reader and the third reader, and 23 (13.0%) were deemed uninterpretable. The remaining 44 videos, not agreed on by the first three readers, were reviewed by the fourth reader. Of these, 28 (63.6%) were interpretable with an RR difference within 2 breaths per minute, and four were deemed uninterpretable between the fourth reader and any of the first three readers. Ultimately, the VEP classified 544 (89.9%) videos as interpretable with an RR difference within 2 breaths per minute, 12 (2.0%) as interpretable with an RR difference greater than 2 breaths per minute and 49 (8.1%) as uninterpretable. The overall interpretation process by the VEP is presented in figure 2.

The interpretability of the videos for assessing RR by the VEP was significantly lower for children aged <12 months compared with older children (p<0.001), and also lower when the child was moving or crying compared with when the child was calm and asleep (p<0.001) (online supplemental tables 1A and B). The agreement in RR within 2 breaths per minute was relatively lower among children who were moving or crying compared with those who were calm or asleep (p<0.001) (online supplemental tables 2A and B).

**Figure 3:**
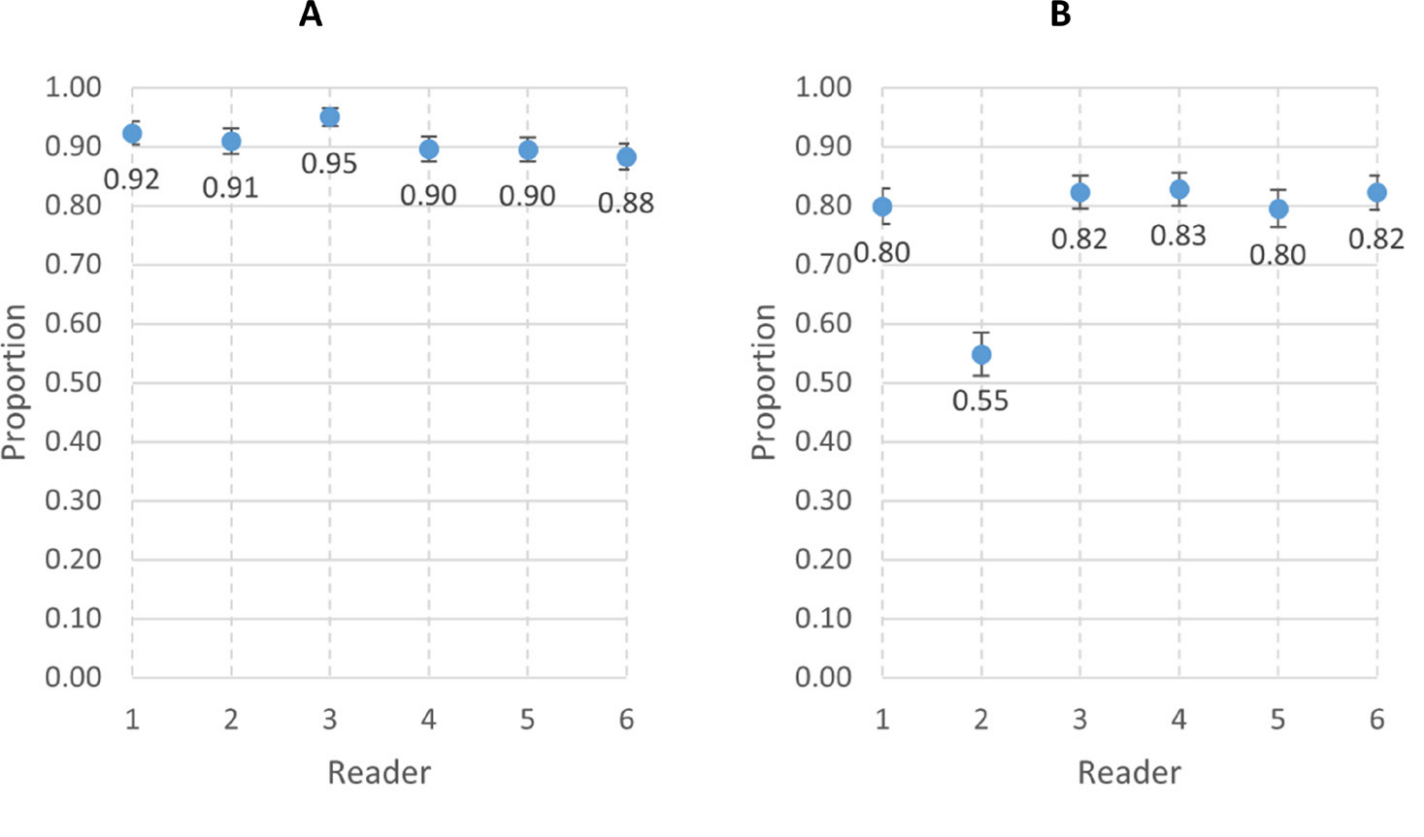
(A) Interobserver agreement for interpretable versus uninterpretable videos among the six primary readers. (B) Interobserver agreement of respiratory rate counts within 2 breaths per minute among the six primary readers.

The individual primary reader inter-reader agreement results for video interpretability and agreement in RR count within 2 breaths per minute are shown in figure 3 and online supplemental tables 3A and B. The interobserver agreement for video interpretability ranged from 88% (reader 6) to 95% (reader 3). For RR measurement, interobserver agreement was 55% for one reader (reader 2), while the remaining readers ranged from 80% (reader 5) to 83% (reader 4).

**Figure 4:**
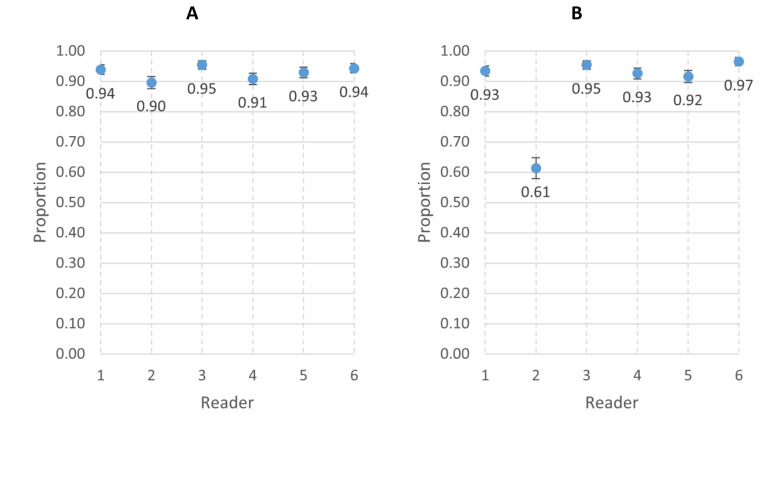
(**A**) Agreement for interpretable versus uninterpretable videos between individual reader and the panel. (**B**) Agreement of respiratory rate counts within two breaths per minute between individual readers and the panel.

Figure 4 and online supplemental tables 4A and B present the individual reader agreement for video interpretability and RR counts compared with the final panel reading. The interpretability agreement ranged from 90% (reader 2) to 95% (reader 3), while the agreement for RR counts was 61% for reader 2 and ranged from 92% (reader 5) to 97% (reader 6) for the other readers.

**Figure 5:**
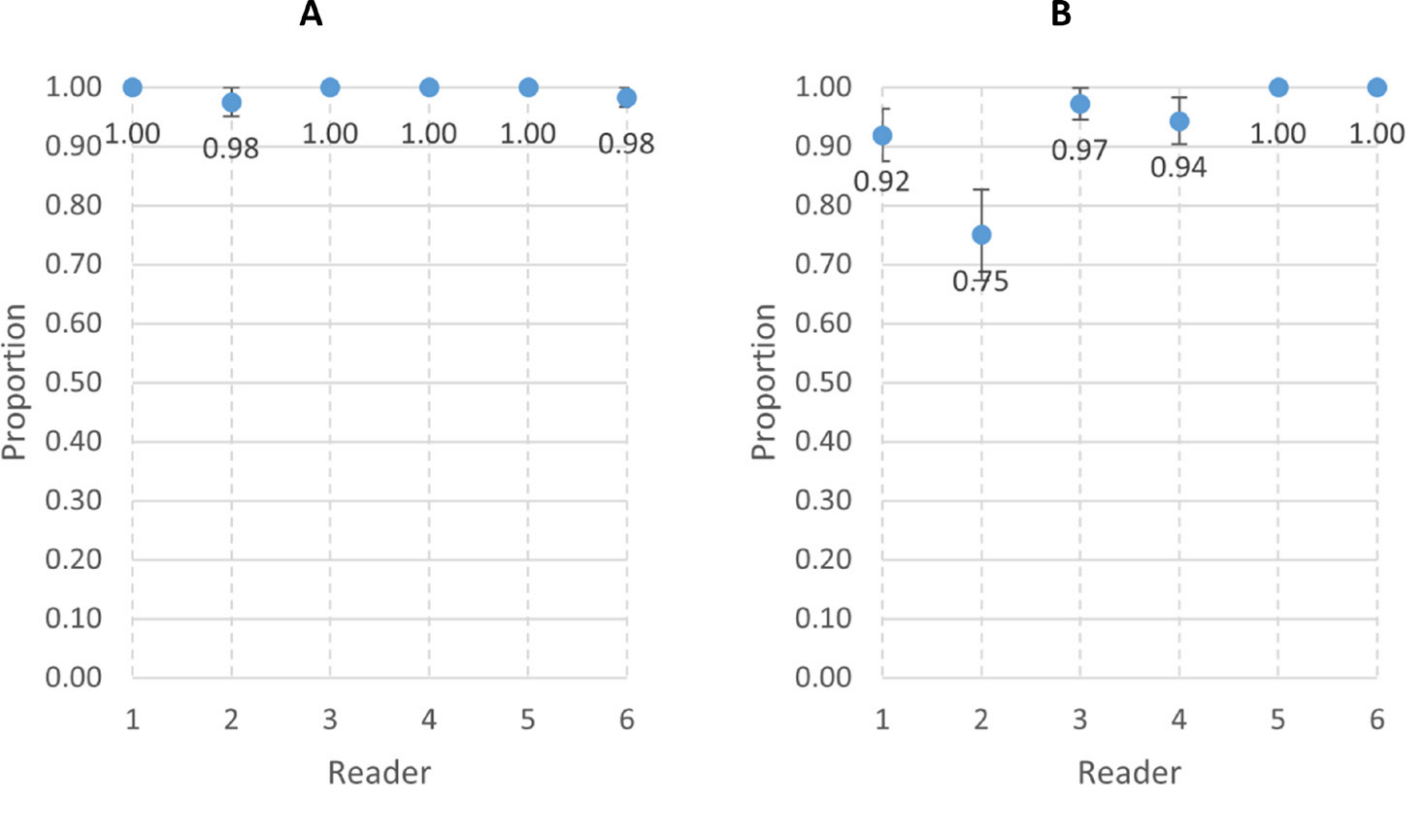
(A) Intrareader agreement for interpretable versus uninterpretable videos in each of the six individual readers. (B) Intrareader agreement of respiratory rate counts within 2 breaths per minute in each of the six individual readers.

Figure 5 and online supplemental tables 5A and B show the intra-reader agreement for video interpretability and RR counts within 2 breaths per minute by individual panel members. Each reader reassessed between 36 and 60 videos. Agreement on video interpretability ranged from 98% (reader 2) to 100% (readers 1, 3, 4 and 5). The agreement for RR counts was 75% for reader 2, while it ranged from 94% (reader 4) to 100% (readers 5 and 6) for the other readers.

54 videos interpreted by the VEP were reviewed by the paediatrician panel. The paediatricians agreed on RR counts within 2 breaths per minute for 53 of the videos. The mean difference in RR counts between the VEP and paediatricians was −0.3 breaths per minute, and the limits of agreement were −3.0–2.3 breaths per minute. The Bland-Altman plot showed that most data points lay within these limits, with no evident trend across the range of mean RR values, indicating strong concordance between the two panels and an absence of systematic bias. These findings suggest that the VEP’s RR assessments were closely aligned with those of the paediatricians across different RRs (online supplemental figure 1). The agreement in RR counts within two breaths per minute between the VEP and paediatrician panel was 88.7%, using the paediatrician panel counts as the gold standard (online supplemental table 6).

To assess the influence of neonatal periodic breathing on agreement, we performed a subanalysis excluding children aged <2 months. In this restricted sample, the overall agreement in RR within 2 breaths per minute between readers improved compared with the full cohort (91.4% vs 89.9%) (online supplemental figure 2).

## Discussion

This study outlined the methodology used by the VEP to evaluate videos for RR measurement and assessed the performance of the VEP both individually and collectively. The performance was evaluated in terms of inter-reader agreement among primary readers, inter-reader agreement between each individual reader and the final panel reading, intrareader agreement for internal consistency and external agreement with paediatricians. The findings demonstrate that the overall performance of the VEP was satisfactory, with five out of six individual panel members performing well.

Currently, there is no established guidance on the optimal methodology for forming and using an expert panel to generate a reference standard.^32^ For the diagnosis of radiographic pneumonia, the WHO has developed standard guidance for forming expert panels and standardising them to interpret chest radiographs for the diagnosis of paediatric pneumonia. This methodology emphasises structured training, calibration and consensus-building among panel members to ensure consistent and reliable interpretation.^41^ Following this guidance, expert panels have been assembled, trained and calibrated using adapted methodologies in various studies focused on diagnosing paediatric radiographic pneumonia.^42^,^44^ A similar approach was taken by Pervaiz *et al*, who developed an expert panel to diagnose paediatric pneumonia using lung ultrasound by adapting the WHO chest radiograph methodology.^45^ Likewise, Ahmed *et al* developed a listening expert panel—also based on WHO guidance—to detect adventitious lung sounds recorded with a digital stethoscope for diagnosing paediatric pneumonia.^46^ Guided by these established methodologies, our study developed a similar VEP consisting of six trained physicians to serve as a reference standard for evaluating RR counting in the diagnosis of pneumonia in children.

Muro *et al* observed significant differences between two paediatricians in RR assessments from video recordings of children under 5 years of age. The paediatricians agreed on RR counts within 2 breaths per minute in 66.9% of the interpretable videos.^28^ In comparison, to improve agreement on RR assessments among readers, our study developed a VEP and reviewed each of the videos with up to four members. Of the 605 videos, the two primary readers agreed on the RR count for 406 videos (67.1%), aligning with the findings of Muro *et al.*^28^ When the two primary readers could not reach an agreement, two additional readers served as adjudicators to resolve discrepancies. The addition of the adjudication level to our work improved overall agreement. The level of agreement improved from 66.9% to 85.3% (516 videos) after involving the third reader (adjudicator #1) and further increased to 89.9% (544 videos) after involving the fourth reader (adjudicator #2). Although the second adjudication round led to only a modest improvement (from 85.5% to 89.9%), it helped confirm borderline or ambiguous cases. It is important to note that higher agreement does not necessarily imply greater accuracy, as readers could agree yet still be incorrect. Only 8.1% of the videos were deemed uninterpretable by the VEP. The use of two adjudicators (third and fourth readers) was pre-specified to ensure additional layers of verification when disagreement persisted after the first and second review rounds. The number was chosen pragmatically to balance data quality assurance with operational feasibility, rather than a predefined statistical threshold of agreement. Our work shows the possible added value of a full panel with adjudicators.

Sinyangwe *et al* also used a VEP to evaluate the performance of community health workers in manually counting RR in children aged <5 years. Each video was sent to a pair of experts who counted RR independently and then compared their readings. Their average count was considered the final RR for that child if the difference in RR count was within 5 breaths per minute between them. Otherwise, both reassessed the video together and reached a consensus. Although the readers were blinded to the results of others during the initial reading, they were not blinded during the consensus stage of adjudication.^29^ Our methods, in comparison, did require masking of readers and we did not include a consensus stage. To ensure the blinding of VEP members’ findings in our study, each member used a password-protected web-based system to interpret RR, and they were also blinded to the clinical findings of the children.

Stratil *et al* developed a panel with 10 medical professionals and conducted an inter-rater reliability study to evaluate the reviewers in counting RR using a video-based reference tool in children under 5 years old. The study revealed high inter-reader agreement among the reviewers. However, the agreement was lower on assessments of younger children.^33^ These findings align with the current study, where the overall agreement between VEP members was high, but the interpretability and agreement were lower in children aged 0–11 months and if the RR was higher. A possible reason for these observations might be that young children usually have a higher RR, making it more difficult to count accurately.

In our study, no gold reference standard was available to evaluate the performance of individual panel members. Instead, their performance was assessed using inter-reader agreement among primary readers, agreement between each reader and the final panel interpretation and intrareader agreement. The inter-reader agreement for video interpretability ranged from 88% to 95%, with most primary readers demonstrating excellent concordance. The agreement in RR counts was more variable: one reader showed relatively poor inter-reader agreement with other primary readers (55%), while the remaining readers achieved satisfactory agreement levels ranging from 80% to 83%. Agreement between individual readers and the final panel reading was notably high, with video interpretability ranging from 90% to 95%, and RR agreement with the final panel ranging from 92% to 97%, except for the outlier reader mentioned above (61%). Each panel member re-evaluated approximately 20% of the videos, and intrareader agreement in RR counts was excellent for five out of six readers, ranging from 92% to 100%. The one low-performing reader had an intra-reader agreement of 75%. These findings demonstrate that it is possible to identify low performance by assessing inter-reader and intrareader agreements, even in the absence of a gold standard. While these findings highlight the robustness of the VEP approach, they also underscore the need for continuous training and calibration among panel members to minimise variability in RR measurement, particularly for readers with lower agreement rates.

Comparison with paediatricians revealed strong alignment between VEP-determined RR and paediatrician-determined RR. Approximately 10% of the randomly selected videos, for which the VEP had reached agreement on RR, were reviewed by paediatricians who independently counted the RR from the same videos. The mean difference in RR between the VEP and the paediatricians was −0.3 breaths per minute, with narrow limits of agreement (95% CI −3.0 to 2.3 breaths per minute). The VEP and paediatricians agreed on RR counts within 2 breaths per minute in 88.7% of the videos, demonstrating very good overall performance of the VEP in this study.

Another important issue is the definition of agreement in RR counts between reviewers. There is variability in how agreement is defined across studies, with thresholds ranging from 2 breaths per minute to five breaths per minute.^12^ In our study, we defined agreement as a difference in RR counts within 2 breaths per minute between two readers, which aligns with the most commonly used definition in existing literature.^23 29 33 39 40^ This threshold is also consistent with the TPP developed by UNICEF, which recommends that new RR counting devices achieve an accuracy of at least 2 breaths per minute compared with a reference standard.^14^

The study has several limitations. First, although the VEP was established following a training and standardisation process, the performance of individual panel members may have deteriorated over time. By the end of the study, some VEP members demonstrated performance that was inferior compared with their peers and to their own earlier performance. The overall panel performance could be improved by periodically evaluating readers after reviewing a certain number of videos and providing remediation or removing those with consistently low performance. Second, there was no gold standard guidance available for evaluating the performance of individual VEP members in this study. Their performance was assessed using inter-reader and intrareader agreement, which, while informative, may not fully capture all aspects of accuracy. Third, the panel’s performance in RR assessments was compared with that of paediatricians, who were treated as the gold standard. However, paediatricians’ measurements cannot be assumed to be universally accurate, introducing potential bias in the comparison. Finally, some children were excluded from the analysis due to lack of cooperation during the assessment. Additionally, about 8% of the videos were deemed uninterpretable by the VEP, primarily due to excessive child movement, and no attempts were made to count RR from these videos. Future research could focus on developing devices capable of consistently measuring RR in non-cooperative children, including young children and those with elevated RR, addressing this critical gap.

In conclusion, this study offers valuable insights into the VEP and suggests that it could serve as an improved reference standard for evaluating RR counting in children with suspected non-severe pneumonia. The VEP demonstrated satisfactory performance in this study. However, further research is required to enhance its performance and validate its use as a reliable reference standard across diverse settings and contexts. The methodology described in this report can be replicated in future studies to assess whether modifications or adaptations are necessary based on specific study environments. As technological advancements continue to emerge in the medical field, the VEP development process must be updated and adapted to integrate new methods of RR counting. This study has laid a solid foundation for future research, which could build on these findings to refine the VEP process and contribute to the development of an ideal reference standard for RR evaluation in children.

## Supplementary material

10.1136/bmjresp-2025-003591 online supplemental file 1

## Data Availability

Data are available upon reasonable request.
