# Supplementary material for: Evaluating the performance of a video expert panel in assessing respiratory rate from recorded videos for the diagnosis of non-severe paediatric pneumonia
Source: BMJ Open Respir Res. 2025 Dec 17;12(1):e003591. doi: 10.1136/bmjresp-2025-003591 (PMC12716567; doi:10.1136/bmjresp-2025-003591)

Evaluating the performance of video expert panel in assessing respiratory rate from recorded videos for paediatric pneumonia diagnosis

Ahad Mahmud Khan, Md Shafiqul Islam, Nabidul Haque Chowdhury, Salahuddin Ahmed, Ting Shi, Abdullah H Baqui, Steve Cunningham, Eric D McCollum, Harry Campbell, RESPIRE Collaboration

**Corresponding author:**

Ahad Mahmud Khan

Projahnmo Research Foundation, Dhaka, Bangladesh

Usher Institute, University of Edinburgh, Edinburgh, UK

Email: ahad_mahmud@hotmail.com

**Procedure for Videography**

**Purpose**

The purpose of this document is to outline the procedures for video recording and editing.

**Scope/Responsibilities**

The document applies to the study staff responsible for video recording activities.

**Equipment**

Video camera, tripod, microphone, stopwatch, Acute respiratory infection (ARI) timer, automated respiratory rate counter (ChARM), an extra battery, an extra SD card, and a computer.

**Software**

Adobe Premiere Pro.

**Mechanic Description**

| Video camera | : | A video camera (e.g., Canon EOS 50) is used to record the video. |
| --- | --- | --- |
| Tripod | : | A tripod is used to mount the camera and stabilize it while recording the video. |
| Microphone | : | A microphone is used to record the starting and ending instructions from the physician or health worker. |
| LED lamp | : | A rechargeable LED lamp is used if optimal lighting is not available in the room. |
| ChARM device | : | ChARM device is used for counting the respiratory rate automatically. |
| ARI timer | : | An ARI timer is used to measure the respiratory rate manually. |
| Extra battery | : | An extra battery is needed to ensure a continuous power supply for the video camera. |
| Extra SD card | : | An extra SD card is needed for storage expansion, if necessary. |
| Computer | : | A computer is required for transferring, storing, and editing videos. |

**Video Recording**

1. **Preparing the video camera**
   1. Turn on the camera by pressing the switch or the on/off button.
   2. Remove the lens cap from the camera lens.
   3. Release the lens so that the camera sensor can see through it.
   4. Ensure that the camera battery is sufficiently charged. If the battery is not fully charged, charge it or use the extra battery.
   5. Ensure that the camera has enough memory space for video recordings. If there is not enough storage, use the extra SD card.
   6. Ensure that the camera’s video settings are suitable for the environment. If the video appears dark or blurred, adjust the settings, such as ISO, aperture, and lens focus.
   7. Mount the camera on a tripod.
      1. Mount the tripod’s baseplate under the camera by rotating it clockwise.
      2. After tightening the baseplate, slowly slide the camera onto the tripod until you hear a clicking sound.
      3. Adjust the height of the tripod to achieve the optimal height and angle for the video.
   8. Connect the microphone to the camera using the microphone port on the camera.
   9. Make sure the video recording environment has optimal lighting.
2. **Recording video**
   1. Ask the caregiver to bring the child to a separate room designated for the video recording procedure. If a separate room is not available or if it is not feasible to move the baby to the room (e.g., if the baby starts crying during the transition, or if the caregiver is unwilling to go to the room), record the video in the current location (e.g., bedside or examination room).
   2. Ask the caregiver to gently and carefully expose the child's chest and abdomen, ensuring that the child remains calm and is not agitated.
   3. Ensure the child is calm before starting the recording. If the child is asleep, record the video as they are (lying down or on the parent’s lap). If the child is awake and under 2 years old, record the video while the child is on the caregiver’s lap or lying down. If the child is 2 years or older, record the video based on the child's preference, either lying down or on the parent’s lap.
   4. Align the video camera horizontally and adjust the tripod to the optimal height and angle. Position the tripod and camera at the side of the child for the best viewing angle. If there is insufficient space to set up the tripod, record the video using a handheld approach.
   5. Ensure the lighting and focus of the video are correct. If optimal lighting is not available, use a rechargeable LED light focused on the child's chest and abdomen.
   6. Once the child is calm, press the record button on the camera to start recording. The physician or health worker will begin counting the respiratory rate using the ARI timer. The physician will count the respiratory rate for one minute and press the ARI timer when the counting is finished. Stop the video recording after hearing the beep from the ARI timer.
   7. After that, secure the ChARM device around the child’s abdomen. Once the child is calm, press the record button on the camera to begin recording. Activate the ChARM device and simultaneously start manually counting the respiratory rate. The video recording will proceed in parallel with the use of the ChARM device, as done previously. Count the total number of breaths until the ChARM device completes its count. The ChARM device will indicate completion by displaying a green or red signal. The video recording should continue until the device completes its process. A maximum of 3 minutes will be allowed for the ChARM device to register a reading or display an error message. If the device fails to do so within this time frame, the attempt will be documented as unsuccessful. Stop the video recording once the ChARM device displays either a green or red signal."
   8. If the video is interrupted due to the child moving or crying, or for any other reason, stop the recording immediately and try again after the child has calmed down or the issue has been resolved. A maximum of three attempts for each manual count will be allowed.
   9. Each video file will be automatically saved on the camera with a file name. Note the video file name from the camera on the data collection form.
3. **Transferring video files to a computer**
   1. Switch off the camera and carefully remove the SD card from it.
   2. Connect the SD card to a computer using a card reader.
   3. Copy the video files from the SD card and paste them into a designated folder on the computer. This process may take some time to complete.
   4. After all the files have been copied, check each video file to ensure none are corrupted.
   5. Once you have confirmed that all files have been copied correctly, delete the files from the SD card to make space for future videos.
   6. Safely remove the card reader from the computer.
   7. Insert the SD card back into the video camera. If the SD card does not appear when the camera is on, turn off the camera, remove the SD card, and reinsert it correctly.
   8. Turn off the camera.
4. **Video editing**
   1. Open Adobe Premiere Pro software on your computer.
   2. Import the video file into the editing software.
      1. In the editing software, find the import option in the dropdown file menu.
      2. Click "Import," navigate to the folder where the videos are stored, select the videos, and click "Open."
   3. First, the video file needs to be trimmed.
      1. Trim the extra portion of the video before the starting indication sound.
      2. Trim the extra portion of the video after the ending indication sound. The length of the video should be exactly 60 seconds.
   4. Extract the audio from the video.
      1. Separate the audio from the video and unlink it.
      2. Delete the extracted audio from the video.
   5. Add a caption to the video.
      1. Use text or graphical content presets to add a caption to the video.
      2. The caption should be the video file name.
      3. Match the caption with the video file name from the enrolment data and save it.
   6. Save the video file after completing the editing process.
   7. Export the video file to a designated folder.
      1. Click the export option in the dropdown file menu.
      2. Rename the video file according to its file name.
      3. Select the destination folder for exporting the video.
      4. Click "OK."
5. **Compressing the video files**

All the original video files have a large file size ranging from 600 MB to 950 MB. Even after editing, the file size of the edited videos ranges from 75 MB to 150 MB. The file size needs to be reduced to 15-30 MB to be uploaded to the system. To compress the video, use an online tool (<https://www.veed.io/tools/video-compressor>) and reduce the file size to meet the required range. After completing the compression process, download the compressed files to a separate folder, using the same name as the original video file.

1. **Upload the videos in the web-based automated system**
   1. Create a folder in the Dropbox or OneDrive Cloud.
   2. Create separate folders for each child.
   3. Copy the compressed videos to the corresponding Child ID folder.
   4. Every folder with a child ID will contain two videos of the same child.
   5. Open the web-based automated system.
   6. Upload the link of each video file according to the child ID.
   7. Close the web-based automated system after linking all the videos at hand.

Supplementary table 1A: Interpretability of respiratory rate by the video expert panel (univariable analysis)

| **Characteristics** | **Interpretability of RR by video expert panel** | | | **p-value** |
| --- | --- | --- | --- | --- |
|  | **Interpretable**  **N (%)** | **Un interpretable**  **N (%)** | **Total**  **N (%)** |  |
| **Age (months)** | | | | |
| <2 | 53 (9.7) | 11 (22.4) | 64 (10.8) | <0.001 |
| 2-11 | 203 (37.3) | 30 (61.2) | 233 (39.3) |  |
| 12-35 | 141 (25.9) | 6 (12.2) | 147 (24.8) |  |
| 16-59 | 147 (27.0) | 2 (4.1) | 149 (25.1) |  |
| **Sex** | | | | |
| Male | 318 (58.5) | 29 (59.2) | 347 (58.5) | 0.921 |
| Female | 226 (41.5) | 20 (40.0) | 246 (41.5) |  |
| **Child condition** | | | | |
| Calm | 270 (49.6) | 9 (18.4) | 279 (47.0) | <0.001 |
| Asleep | 222 (40.8) | 3 (6.1) | 225 (37.9) |  |
| Moving | 49 (9.0) | 36 (73.5) | 85 (14.3) |  |
| Crying | 3 (0.6) | 1 (2.0) | 4 (0.7) |  |
| **Health facility** | | | | |
| ICMH | 172 (31.6) | 10 (20.4) | 182 (30.7) | 0.009 |
| UHC | 172 (31.6) | 26 (53.1) | 198 (33.4) |  |
| CC | 200 (36.8) | 13 (26.5) | 213 (35.9) |  |
| **Assessing healthcare staff** | | | | |
| Physician | 344 (63.2) | 36 (73.5) | 380 (64.1) | 0.153 |
| CHCP | 200 (36.8) | 13 (26.5) | 213 (35.9) |  |
| **Total** | **544 (100)** | **49 (100)** | **593 (100)** |  |

CC – Community clinic, CHCP – Community Health Care Provider, ICMH – Institute of Child and Mother Health, UHC – Upazila Health Complex

Supplementary table IB: Interpretability of respiratory rate by the video expert panel (multivariable analysis)

| **Factors** | **Unadjusted OR (95% CI)** | **p-value** | **Adjusted OR (95% CI)** | **p-value** |
| --- | --- | --- | --- | --- |
| **Age (months)** | | | | |
| <2 | Ref |  | Ref |  |
| 2-11 | 1.4 (0.6 – 3.1) | 0.406 | 1.4 (0.6 – 3.5) | 0.492 |
| 12-35 | 4.9 (1.7 – 14.0) | 0.003 | 4.5 (1.4 – 15.0) | 0.012 |
| 16-59 | 15.3 (3.2 – 71.7) | 0.001 | 7.8 (1.3 – 47.0) | 0.025 |
| **Sex** | | | | |
| Male | 1.0 (0.5 – 1.8) | 0.925 |  |  |
| Female | Ref |  |  |  |
| **Child condition** | | | | |
| Calm or asleep | 29.2 (14.8 – 57.5) | <0.001 | 20.0 (9.4 – 41.0) | <0.001 |
| Moving or crying | Ref |  | Ref |  |
| **Health facility** | | | | |
| ICMH | 1.1 (0.5 – 2.8) | 0.810 | 2.9 (1.0 – 8.3) | 0.053 |
| UHC | 0.4 (0.2 – 0.9) | 0.0.25 | 1.5 (0.6 – 3.3) | 0.378 |
| CC | Ref |  | Ref |  |
| **Assessing healthcare staff** | | | | |
| Physician | 0.6 (0.3 – 1.2) | 0.181 |  |  |
| CHCP | Ref |  |  |  |

bpm – breaths per minute, CC – Community clinic, CHCP – Community Health Care Provider, CI – Confidence Interval, ICMH – Institute of Child and Mother Health, OR – Odds Ratio, RR – Respiratory rate, UHC – Upazila Health Complex

Supplementary table 2A: Agreement in respiratory rate counts within 2 bpm among the interpretable videos by the video expert panel (univariable analysis)

| **Characteristics** | **Agreement in RR count by VEP** | | | **p-value** |
| --- | --- | --- | --- | --- |
|  | **Yes**  **N (%)** | **No**  **N (%)** | **Total**  **N (%)** |  |
| **Age (months)** | | | | |
| <2 | 53 (9.7) | 4 (33.3) | 57 (10.3) | 0.013 |
| 2-11 | 203 (37.3) | 6 (50.0) | 209 (37.6) |  |
| 12-35 | 141 (25.9) | 0 (0.0) | 141 (25.4) |  |
| 16-59 | 147 (27.0) | 2 (16.7) | 149 (26.8) |  |
| **Sex** | | | | |
| Male | 318 (58.5) | 11 (91.7) | 329 (59.2) | 0.033 |
| Female | 226 (41.5) | 1 (8.3) | 227 (40.8) |  |
| **Child condition** | | | | |
| Calm | 270 (49.6) | 2 (16.7) | 272 (48.9) | <0.001 |
| Asleep | 222 (40.8) | 1 (8.3) | 223 (40.1) |  |
| Moving | 49 (9.0) | 9 (75.0) | 58 (10.4) |  |
| Crying | 3 (0.6) | 0 (0.0) | 3 (0.5) |  |
| **Health facility** | | | | |
| ICMH | 172 (31.6) | 1 (8.3) | 173 (31.1) | 0.031 |
| UHC | 172 (31.6) | 8 (66.7) | 180 (32.4) |  |
| CC | 200 (36.8) | 3 (25.0) | 203 (36.5) |  |
| **Assessing healthcare staff** | | | | |
| Physician | 344 (63.2) | 9 (75.0) | 353 (63.5) | 0.550 |
| CHCP | 200 (36.8) | 3 (25.0) | 203 (36.5) |  |
| **RR interpreted by VEP (bpm)** | | | | |
| <40 | 329 (60.5) | 3 (25.0) | 332 (59.7) | 0.021 |
| 40-59 | 158 (29.0) | 6 (50.0) | 164 (29.5) |  |
| ≥60 | 57 (10.5) | 3 (25.0) | 60 (10.8) |  |
| **Total** | **544 (100)** | **12 (100)** | **556 (100)** |  |

bpm – breaths per minute, CC – Community clinic, CHCP – Community Health Care Provider, ICMH – Institute of Child and Mother Health, UHC – Upazila Health Complex

**Supplementary table 2B: Agreement in respiratory rate counts within 2 bpm among the interpretable videos by the video expert panel (multivariable analysis)**

| **Factors** | **Unadjusted OR (95% CI)** | **p-value** | **Adjusted OR (95% CI)** | **p-value** |
| --- | --- | --- | --- | --- |
| **Age (months)** | | | | |
| <2 | Ref |  |  |  |
| 2-11 | 2.6 (0.6 – 10.3) | 0.187 |  |  |
| 12-35 | - | - |  |  |
| 16-59 | 5.5 (1.0 – 31.3) | 0.052 |  |  |
| **Sex** | | | | |
| Male | 0.1 (0.0 – 1.0) | 0.052 | 0.14 (0.0 – 1.1) | 0.065 |
| Female | Ref |  | Ref |  |
| **Child condition** | | | | |
| Calm or asleep | 28.4 (8.4 – 95.6) | <0.001 | 19.0 (4.4 – 78.0) | <0.001 |
| Moving or crying | Ref |  | Ref |  |
| **Health facility** | | | | |
| ICMH | 2.6 (0.3 – 25.1) | 0.414 | 5.8 (0.6 – 60.0) | 0.139 |
| UHC | 0.3 (0.1 – 1.3) | 0.109 | 1.0 (0.2 – 4.9) | 0.953 |
| CC | Ref |  | Ref |  |
| **Assessing healthcare staff** | | | | |
| Physician | 0.6 (0.1 – 2.2) | 0.419 |  |  |
| CHCP | Ref |  |  |  |
| **RR interpreted by VEP (bpm)** | | | | |
| <40 | 5.8 (1.1 – 29.7) | 0.036 | 4.2 (0.6 – 30.0) | 0.156 |
| 40-59 | 1.4 (0.3 – 6.4) | 0.674 | 1.4 (0.2 – 9.2) | 0.719 |
| ≥60 | Ref |  | Ref |  |

bpm – breaths per minute, CC – Community clinic, CHCP – Community Health Care Provider, CI – Confidence Interval, ICMH – Institute of Child and Mother Health, OR – Odds Ratio, RR – Respiratory rate, UHC – Upazila Health Complex

Supplementary table 3A: Interobserver agreement for interpretable versus uninterpretable videos among the six primary readers

| **Reader** | **Number of videos reviewed for assessing interpretability** | **Number of videos in agreement with other readers** | **Proportion of videos in agreement with other readers** | **Standard error** | **Lower confidence interval** | **Upper confidence interval** |
| --- | --- | --- | --- | --- | --- | --- |
| Reader 1 | 194 | 179 | 0.923 | 0.019 | 0.876 | 0.956 |
| Reader 2 | 197 | 179 | 0.909 | 0.021 | 0.859 | 0.945 |
| Reader 3 | 202 | 192 | 0.950 | 0.015 | 0.911 | 0.976 |
| Reader 4 | 202 | 181 | 0.896 | 0.021 | 0.845 | 0.934 |
| Reader 5 | 209 | 187 | 0.895 | 0.021 | 0.845 | 0.933 |
| Reader 6 | 206 | 182 | 0.883 | 0.022 | 0.832 | 0.924 |

**Supplementary table 3B: Interobserver agreement of RR counts within 2 bpm among the six primary readers**

| **Reader** | **Number of videos in agreement for interpretability with other readers** | **Number of interpretable videos reviewed for RR counting** | **Number of videos in agreement with other readers** | **Proportion of videos in agreement with other readers** | **Standard error** | **Lower confidence interval** | **Upper confidence interval** |
| --- | --- | --- | --- | --- | --- | --- | --- |
| Reader 1 | 179 | 174 | 139 | 0.799 | 0.030 | 0.732 | 0.856 |
| Reader 2 | 179 | 177 | 97 | 0.548 | 0.037 | 0.472 | 0.623 |
| Reader 3 | 192 | 185 | 152 | 0.822 | 0.028 | 0.759 | 0.874 |
| Reader 4 | 181 | 180 | 149 | 0.828 | 0.028 | 0.765 | 0.880 |
| Reader 5 | 187 | 171 | 136 | 0.795 | 0.031 | 0.727 | 0.853 |
| Reader 6 | 182 | 169 | 139 | 0.822 | 0.029 | 0.756 | 0.877 |

**Supplementary table 4A: Agreement for interpretable versus uninterpretable videos between individual reader and the panel**

| **Reader** | **Number of videos reviewed for assessing interpretability** | **Number of videos in agreement with the panel** | **Proportion of videos in agreement with the panel** | **Standard error** | **Lower confidence interval** | **Upper confidence interval** |
| --- | --- | --- | --- | --- | --- | --- |
| Reader 1 | 241 | 226 | 0.938 | 0.016 | 0.899 | 0.965 |
| Reader 2 | 230 | 206 | 0.896 | 0.020 | 0.849 | 0.932 |
| Reader 3 | 239 | 228 | 0.954 | 0.014 | 0.919 | 0.977 |
| Reader 4 | 238 | 216 | 0.908 | 0.019 | 0.863 | 0.941 |
| Reader 5 | 240 | 223 | 0.929 | 0.017 | 0.889 | 0.958 |
| Reader 6 | 245 | 231 | 0.943 | 0.015 | 0.906 | 0.968 |

**Supplementary table 4B: Agreement of RR counts within 2 bpm between individual readers and the panel**

| **Reader** | **Number of videos in agreement for interpretability with the panel** | **Number of interpretable videos reviewed for RR counting** | **Number of videos in agreement with the panel** | **Proportion of videos in agreement with the panel** | **Standard error** | **Lower confidence interval** | **Upper confidence interval** |
| --- | --- | --- | --- | --- | --- | --- | --- |
| Reader 1 | 226 | 212 | 198 | 0.934 | 0.017 | 0.892 | 0.963 |
| Reader 2 | 206 | 191 | 117 | 0.613 | 0.035 | 0.540 | 0.682 |
| Reader 3 | 228 | 216 | 206 | 0.954 | 0.014 | 0.917 | 0.978 |
| Reader 4 | 216 | 215 | 199 | 0.926 | 0.018 | 0.882 | 0.957 |
| Reader 5 | 223 | 190 | 174 | 0.916 | 0.020 | 0.867 | 0.951 |
| Reader 6 | 231 | 202 | 195 | 0.965 | 0.013 | 0.930 | 0.986 |

**Supplementary table 5A: Intra-reader agreement for interpretable versus uninterpretable videos in each of the six individual readers**

| **Reader** | **Number of videos reviewed for assessing interpretability** | **Number of videos in agreement during subsequent assessment** | **Proportion of videos in agreement during subsequent assessment** | **Standard error** | **Lower confidence interval** | **Upper confidence interval** |
| --- | --- | --- | --- | --- | --- | --- |
| Reader 1 | 40 | 40 | 1.000 | 0.000 | 0.912 | 1.000 |
| Reader 2 | 40 | 39 | 0.975 | 0.025 | 0.868 | 0.999 |
| Reader 3 | 37 | 37 | 1.000 | 0.000 | 0.905 | 1.000 |
| Reader 4 | 36 | 36 | 1.000 | 0.000 | 0.903 | 1.000 |
| Reader 5 | 43 | 43 | 1.000 | 0.000 | 0.918 | 1.000 |
| Reader 6 | 60 | 59 | 0.983 | 0.017 | 0.911 | 1.000 |

**Supplementary table 5B: Intra-reader agreement of RR counts within 2 bpm in each of the six individual readers**

| **Reader** | **Number of videos in agreement for interpretability during reassessment** | **Number of interpretable videos reviewed for RR counting** | **Number of videos in agreement during reassessment** | **Proportion of videos in agreement during reassessment** | **Standard error** | **Lower confidence interval** | **Upper confidence interval** |
| --- | --- | --- | --- | --- | --- | --- | --- |
| Reader 1 | 40 | 37 | 34 | 0.919 | 0.045 | 0.781 | 0.983 |
| Reader 2 | 39 | 32 | 24 | 0.750 | 0.077 | 0.566 | 0.885 |
| Reader 3 | 37 | 36 | 35 | 0.972 | 0.027 | 0.855 | 0.999 |
| Reader 4 | 36 | 35 | 33 | 0.943 | 0.039 | 0.808 | 0.993 |
| Reader 5 | 43 | 34 | 34 | 1.000 | 0.000 | 0.897 | 1.000 |
| Reader 6 | 59 | 49 | 49 | 1.000 | 0.000 | 0.927 | 1.000 |

**Supplementary figure 1 Bland Altman plot showing the agreement between video expert panel respiratory rate counts and paediatrician respiratory rate counts**


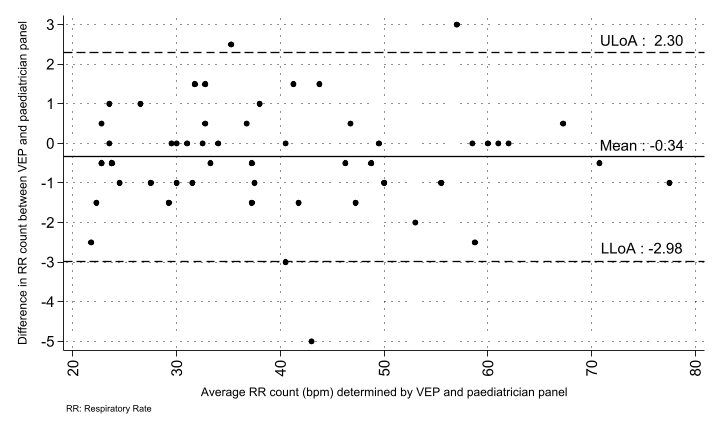


**Supplementary table 6 Agreement in respiratory rate count by video expert panel and paediatricians (gold standard)**

| Respiratory rate (bpm) | Agreement within 2 bpm  **n/N (%)** |
| --- | --- |
| <40 | 28/31 (90.3) |
| 40-49 | 10/11 (90.9) |
| 50-59 | 3/5 (60.0) |
| ≥60 | 6/6 (100.0) |
| Total | 47/53 (88.7) |

bpm – breaths per minute

**Supplementary figure 2: Chest video image interpretation schema (children aged 2-59 months)**


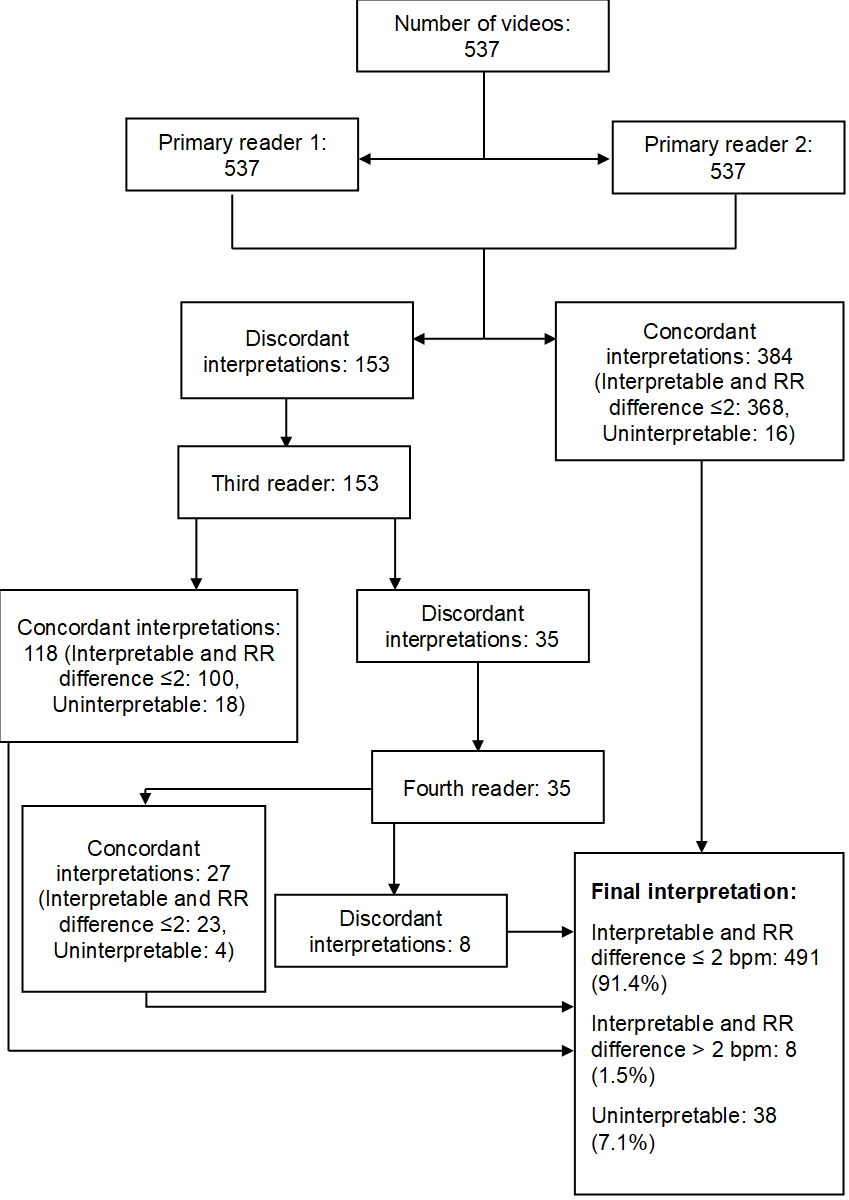

Supplement: online supplemental file 1 [file bmjresp-12-1-s001.docx]
